# The Role of the Conjugate Bridge in Electronic Structures and Related Properties of Tetrahydroquinoline for Dye Sensitized Solar Cells

**DOI:** 10.3390/ijms14035461

**Published:** 2013-03-08

**Authors:** Cai-Rong Zhang, Li Liu, Jian-Wu Zhe, Neng-Zhi Jin, Yao Ma, Li-Hua Yuan, Mei-Lin Zhang, You-Zhi Wu, Zi-Jiang Liu, Hong-Shan Chen

**Affiliations:** 1 State Key Laboratory of Gansu Advanced Non-ferrous Metal Materials, Lanzhou University of Technology, Lanzhou 730050, Gansu, China; E-Mails: wangyezhizuohao@126.com (L.L.); youzhiwu@163.com (Y.-Z.W.); 2 Department of Applied Physics, Lanzhou University of Technology, Lanzhou 730050, Gansu, China; E-Mails: yuanlh@lut.cn (L.-H.Y.); zhangml_2000@126.com (M.-L.Z.); 3 Gansu Computing Center, Lanzhou 730030, Gansu, China; E-Mails: zjw@gspcc.com (J.-W.Z.); jin_n_z@163.com (N.-Z.J.); may@gspcc.com (Y.M.); 4 Institute of Electronic Information Science and Technology, Lanzhou City University, Lanzhou 730070, Gansu, China; E-Mail: liuzj_scu@126.com; 5 College of Physics and Electronic Engineering, Northwest Normal University, Lanzhou 730070, Gansu, China; E-Mail: chenhs@nwnu.edu.cn

**Keywords:** tetrahydroquinoline dyes, electronic structure, density functional theory, absorption spectra, dye sensitized solar cells

## Abstract

To understand the role of the conjugate bridge in modifying the properties of organic dye sensitizers in solar cells, the computations of the geometries and electronic structures for 10 kinds of tetrahydroquinoline dyes were performed using density functional theory (DFT), and the electronic absorption and fluorescence properties were investigated via time dependent DFT. The population analysis, molecular orbital energies, radiative lifetimes, exciton binding energies (EBE), and light harvesting efficiencies (LHE), as well as the free energy changes of electron injection (Δ*G^inject^* ) and dye regeneration ( $\Delta G_{\mathrm{dye}}^{\mathrm{regen}}$ ) were also addressed. The correlation of charge populations and experimental open-circuit voltage (*V*_oc_) indicates that more charges populated in acceptor groups correspond to larger *V*_oc_. The elongating of conjugate bridge by thiophene units generates the larger oscillator strength, higher LHE, larger absolute value of Δ*G^inject^*, and longer relative radiative lifetime, but it induces the decreasing of EBE and $\Delta G_{\mathrm{dye}}^{\mathrm{regen}}$. So the extending of conjugate bridge with thiopene units in organic dye is an effective way to increase the harvest of solar light, and it is also favorable for electron injection due to their larger Δ*G^inject^*. While the inversely correlated relationship between EBE and LHE implies that the dyes with lower EBE produce more efficient light harvesting.

## 1. Introduction

As a novel photovoltaic device, dye sensitized solar cells (DSC) have attracted much attention because of many merits, such as easy fabrication, lower cost, *etc.*[1–5]. However, the photo-to-current conversion efficiency (PCE) of DSC is still not high enough to commercialize. The central interest of researching DSC is how to improve the PCE. It has been found that all of the main components of DSC, including dye sensitizers, anode, and cathode, as well as electrolyte, can affect the PCE. Particularly, the dye sensitizers, which take the function of light harvesting and photo-excited electron injection in DSC, have a significant influence on the PCE [6–11].

Up to now, the dye sensitizers of DSC can be classified into metal-organic complexes and metal-free organic dyes [7–10]. In metal-free organic dyes sensitizers, such as cyanines [12–14], hemicyanines [15,16], triphenylmethanes [8,17,18], perylenes [19–23], coumarins [24–26], porphyrins [27–32], squaraines [33–35], indoline [36,37], and azulene-based dyes [38]*etc.*, were developed since their high molar absorption coefficient, relatively simple synthetic procedure, various structures, and lower cost. Recently, the 12.3% PCE has been achieved by cosensitization of donor-π conjugate bridge-acceptor (D-π-A) zinc porphyrin dye YD2-o-C8 with another triphenylamine-based organic dye Y123 [39]. So the development of novel organic dye sensitizer is a promising method to improve the PCE of DSC.

For dye sensitizer with good performance in DSCs, the following properties are required [40]. First, the dye should have a broad absorption spectrum in the visible region to harvest more photons from solar radiation. Therefore, the wide absorption region and larger absorption coefficient are expected. Second, in terms of simplified schematic of the energetic processes occurring in conventional DSC and weak coupling limit, the dye should have the suitable energy level of ground state S_0_ and excited state S_1_. The level of S_0_ must locate below the redox couple of electrolyte, and therefore the electron acceptance of oxidized dye from electrolyte is energetically permitted. The level of S_1_ should be suitable above the conduction band of semiconductor in anode so that the electron in excited dyes can inject into the anode semiconductor. Also, the S_1_ should have significant charge transfer (CT) character. So, the further developments of novel dye sensitizers depend on the quantitative information of dye sensitizers [9,11,41], such as electronic structures, absorption properties, *etc.* The theoretical investigations of the physical properties of dye sensitizers pave the way to disclose the relationships among the performance, structures, and the properties. For instance, on the basis of density functional theory (DFT) calculations for the chemical structures and exciton binding energies (EBE) of several pure organic dyes, the novel organic triphenylamine-based dye sensitizer EB-01 was designed, and the over 9% of PCE was achieved by EB-01 sensitized DSC [42].

The metal-free organic dye sensitizers with good performance in DSC usually have a D-π-A structure [7–10]. In D-π-A dyes, the π-electron delocalization from the electron-donor to the electron-acceptor through the π-conjugated linker can affect the performance of DSC [43–45]. Therefore, the π-conjugated linker should play an essential role in CT process and photophysical property in the D-π-A dyes. The tetrahydroquinoline dyes had been engineered and synthesized as sensitizers for applications in DSC, and the different length of thiophene-containing conjugate bridges were introduced to dye sensitizers and served as electron spacers [46,47]. To understand the role of conjugate bridge in modifying the properties of organic dye sensitizers in solar cells, the computations of the geometries and electronic structures for 10 kinds of tetrahydroquinoline dyes (the molecular structures presented in Table 1) were performed using DFT, and the electronic absorption and fluorescence properties were investigated via time dependent DFT (TDDFT) with polarizable continuum model (PCM) for solvent effects. The population analysis, molecular orbital energies, radiative lifetimes, EBE, light harvesting efficiencies (LHE), and the free energy changes of electron injection ( Δ*G^inject^* ) and dye regeneration ( $\Delta G_{\mathrm{dye}}^{\mathrm{regen}}$ ) are also studied.

## 2. Computational Methods

The accuracy of DFT calculations depend on the selected functional and basis sets. It has been demonstrated that the polarized split-valence 6-31G(d,p) basis sets are sufficient for calculating the excitation properties of organic dyes [48], and introducing additional diffuse functions in basis sets generate negligible effects on the electron density and hence on the accuracy of DFT and TDDFT results [49]. So the 6-31G(d,p) basis sets were adopted in the present work. Generally, for the dye sensitizers with good performance in DSC, the electronic excitations are CT processes. To select functional for the reliable description of excited properties of dyes, the dye sensitizer C1-1 was selected as the representative dye. The geometry in gas phase was optimized, and then it was further optimized in solvent. Based upon the optimized geometry in solvent, the absorption properties were investigated using TDDFT. The non-equilibrium version of the PCM [50,51] was adopted for calculating the solvent effects. The above mentioned calculations were performed with different functionals, including the hybrid functionals PBE0 [52–54], M062X [55], and the long range corrected hybrid functionals CAM-B3LYP [56], LC-ωPBE [57–60], and ωB97X [61]. Table 2 presents the comparison between calculated λ_max_ using different functionals and experimental λ_max_ for C1-1. The discrepancy between the experimental and calculated results with PBE0, CAM-B3LYP, LC-ωPBE, ωB97X, and M062X functionals are about 0.47, 0.02, 0.49, 0.34, and 0.03 eV, respectively. Apparently, the CAM-B3LYP and M062X functionals provide reliable results, predicting excitation energies agree well with the experimental data. Furthermore, the absolute deviation of CAM-B3LYP functional is slightly smaller than that of M062X. Therefore, CAM-B3LYP is the selected functional for calculating the geometries, electronic structures, electronic absorption, as well as fluorescence spectrum for other tetrahydroquinoline dyes. The solvent effects were also dealt with PCM method. All of the calculations were performed by using Gaussian09 package [62].

## 3. Results and Discussion

### 3.1. Geometrical Structures

**Figure 1:**
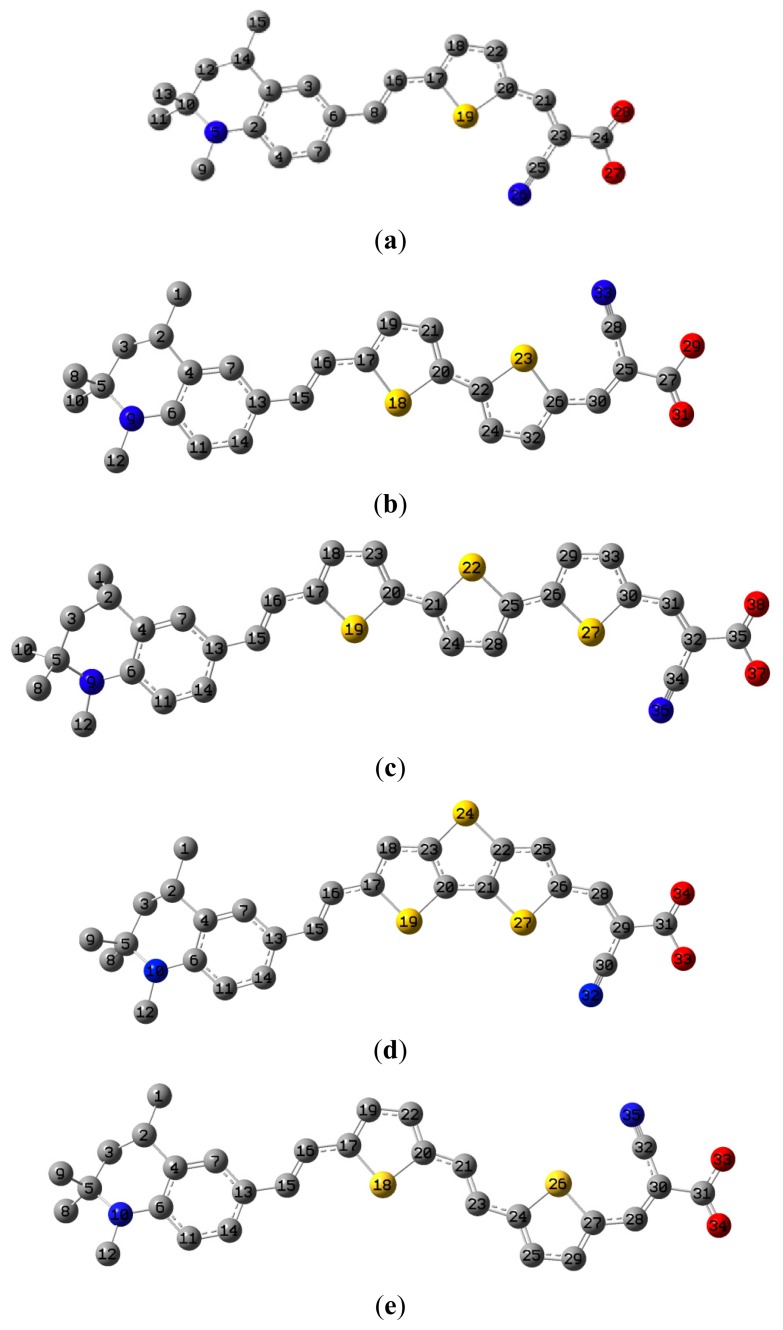
The optimized geometrical structures of dyes (**a**) C1-1, (**b**) C1-2, (**c**) C1-3, (**d**) C1-4, and (**e**) C1-5. (CAM-B3LYP/6-31G(d,p) in gas phase, the blue colored spheres: N; the red colored spheres: O; the yellow colored spheres: S; the other dark colored spheres: C; the hydrogen atoms have been omitted for clarity.)

**Figure 2:**
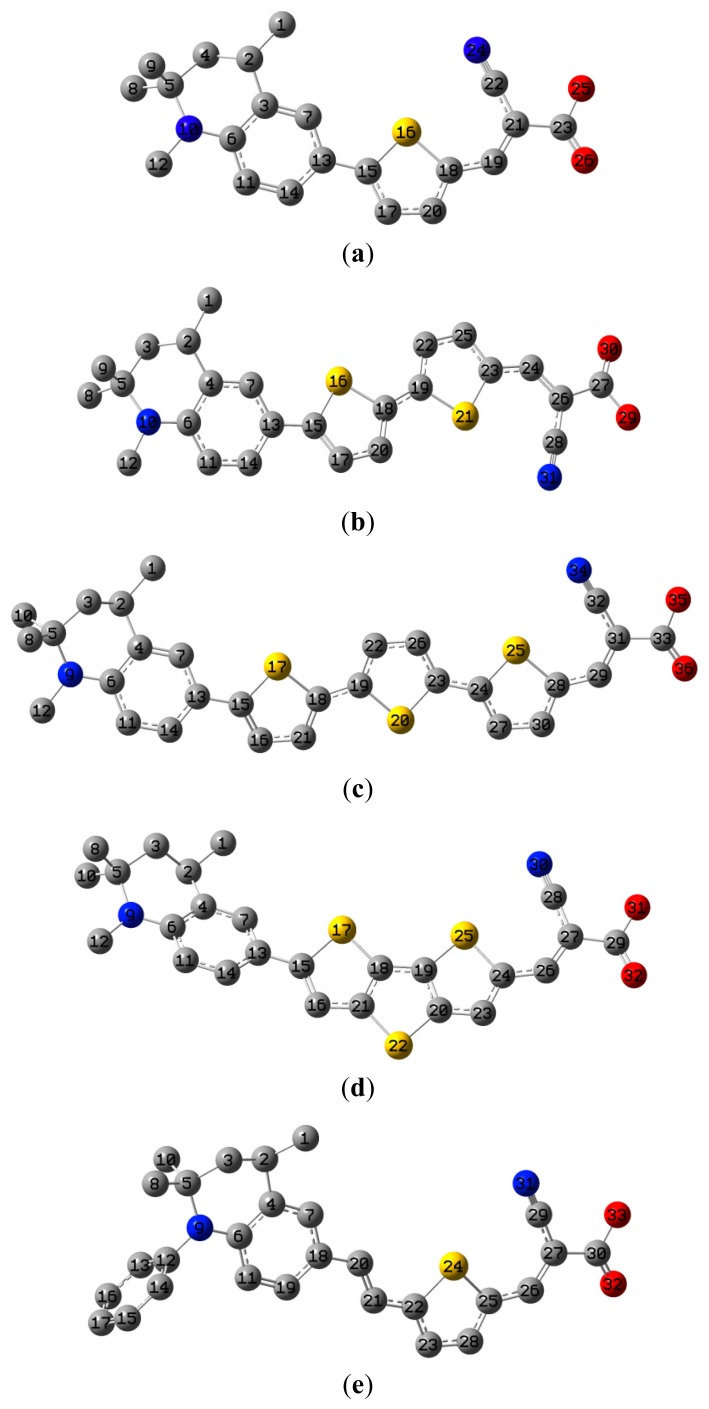
The optimized geometrical structures of dyes (**a**) C2-1, (**b**) C2-2, (**c**) C2-3, (**d**) C2-4, and (**e**) C3-1. (CAM-B3LYP/6-31G(d,p) in gas phase, the blue colored spheres: N; the red colored spheres: O; the yellow colored spheres: S; the other dark colored spheres: C; the hydrogen atoms have been omitted for clarity.)

The optimized geometries of tetrahydroquinoline dyes C1-1, C1-2, C1-3, C1-4, C1-5, C2-1, C2-2, C2-3, C2-4, and C3-1 are shown in Figures 1 and 2. The selected geometrical parameters, including bond lengths, bond angles, and dihedrals are listed in Tables S1–S3 in supporting information (SI). The calculated geometrical data indicate that the geometrical parameters of same group (tetrahydroquinoline, vinylene, thienyl and dithieno[3,2-b;2′,3′-d]thienyl, *etc.*) in different dyes are very similar. This can be understood from the localized interaction of chemical bonds. The cyanoacrylic acid groups in the dyes are coplanar with the groups in conjugate bridge. The torsion angles among the thiophene-rings in bithiophene and dithieno-[3,2-b;2′,3′-d]thiophene groups are quite small. For instance, the torsion angle between thiophene-rings in bithiophene in C1-2 is about 1.7°. The conjugated effects, which can be enhanced by the quasi-planar structure of bridge and the coplanar character between acceptor moieties and conjugate bridges, extend the delocalization of electron, and it is favorable for efficient CT from the chromophore to the carboxyl group. Similar geometric characters are also appeared in the organic dye sensitizers JK-1 and JK-2 [63], JK-16 and JK-17 [64,65], and other organic dyes which contain cyanoacrylic acid group [66–69].

**Figure 3:**
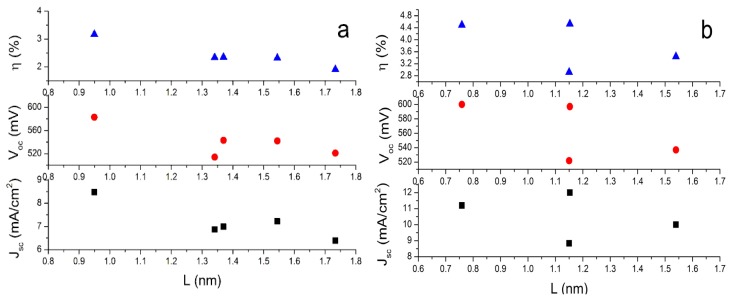
The open-circuit photovoltage (*V*_oc_), short-circuit photocurrent density (*J*_sc_), and solar-to-electrical energy conversion efficiencies (η) *versus* the length of conjugate bridges. (**a**) C1-*n* dyes, *n* = 1–5; (**b**) C2-*m* dyes, *m* = 1–4.

To investigate the role of conjugate bridge length in the performance of dyes in DSCs, we define “*L*” as the distance between two special C atoms. One is the C atom in the carboxyl group. The other one is the C atom in tetrahydroquinoline moiety where the conjugate bridge connects. When the dyes adsorbed on the surface of semiconductor nanoparticles, the photo-induced CT occurs at the interface between dyes and semiconductor nanoparticles. Thus, the *L* can describe the CT distance and it can describe conjugate length to some extent. The Table S4 in SI lists the calculated *L*. The data indicate that the lengths of conjugate bridge with bithiophene and dithieno-[3,2-b;2′,3′-d]thiophene groups are very similar. On the basis of the calculated *L* and the reported photovoltaic experimental data [47], the dependence of open-circuit photovoltage (*V*_oc_), short-circuit photocurrent density (*J*_sc_), and solar-to-electrical energy conversion efficiencies (η) on the length of conjugate bridges are plotted in Figure 3. It can be found that the longest *L* with terthiophene is unfavorable to improve dye performance in DSCs, while the shorter *L* with thiophene (C2-1) or thienylvinyl (C1-1) have better performance. It is understandable because the longer *L* may increase the possibility that the excited electrons decay to the electrolyte [70].

### 3.2. Electronic Structures

In order to analyze the charge distribution and the electron-transfer mechanism of D-π-A dyes, the Natural Bond Orbital (NBO) analysis has been performed, based upon the optimized structure of the ground state. The calculated NBO results are listed in Table 3. The positive charges of tetrahydroquinoline moieties represent the acts as an effective electron-donor unit. Contrarily, the negative NBO charges of cyanoacrylic acid reveal that the electrons trap in the electron-acceptor unit. The data also suggest the charges of similar acceptors in different dyes are very similar, but the charges of donors and conjugate bridges are more flexible. This means that the natural charges in dye sensitizers are dominated by acceptor moieties.

**Figure 4:**
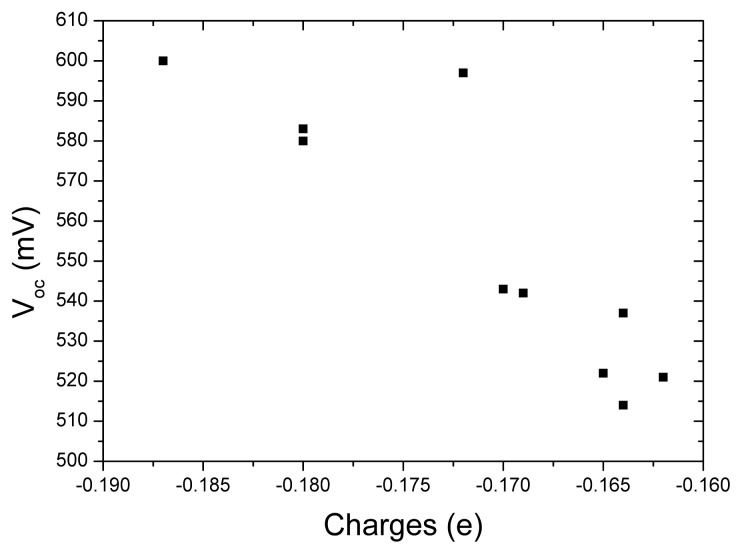
The open-circuit voltage *V*_oc_*versus* the charges of acceptor groups in tetrahydroquinoline dyes.

The open-circuit voltage *V*_oc_*versus* the charges of acceptor groups in tetrahydroquinoline dyes is presented in Figure 4 in order to investigate the relationship between these quantities. It can be found that more charges populated in acceptor groups correspond to larger *V*_oc_. This means more charges populated in acceptor are favorable to increase *V*_oc_. So, introducing strong electron-withdrawing group in acceptor of dye sensitizer is a possible way to increase *V*_oc_ of DSC. This tendency is similar to our previous work for the dyes of coumarin NKX derivatives [70].

**Figure 5:**
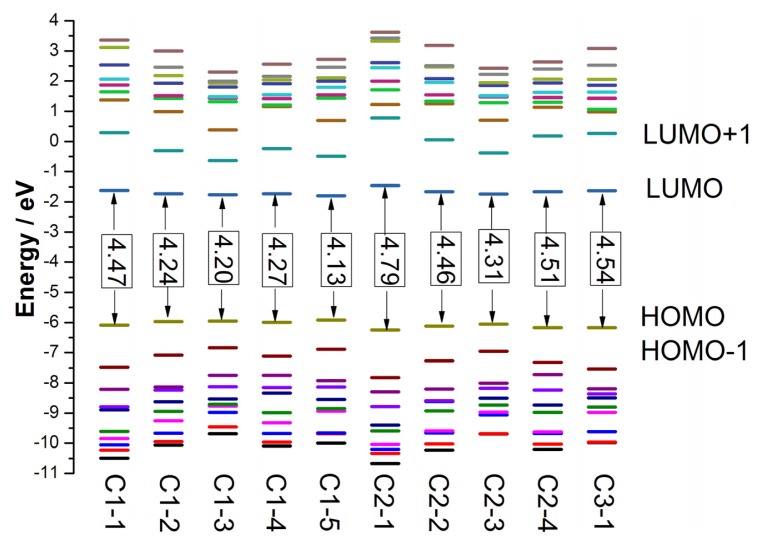
The calculated frontier molecular orbital energies of tetrahydroquinoline dyes (CAM-B3LYP/6-31G(d,p)).

The highest occupied molecular orbital (HOMO) and the lowest unoccupied molecular orbital (LUMO) energies of better dye sensitizers are required to locate suitable values for matching the conduction band edge of semiconductor and redox potential of electrolyte in DSCs. The HOMO level corresponds to the oxidation potential of dye sensitizer [71], and the larger oxidation potential increase the driving force for the reduction of oxidized dye [72]. The HOMO and LUMO energies, as well as the HOMO-LUMO gaps (*E*_g_) of tetrahydroquinoline dyes in solvent are presented in Figure 5, and the data are listed in Table S5 in SI. The ranges of HOMO, LUMO, and *E*_g_ for tetrahydroquinoline dyes in gas phase are about −5.85 to −6.26 eV, −1.32 to −1.77 eV and 4.09 to 4.94 eV, respectively, and the corresponding data in solvent are about −5.92 to −6.25 eV, −1.46 to −1.79 eV and 4.13 to 4.79 eV, respectively. For C1-*n* and C2-*n* (*n* = 1–3) dyes, the HOMO energies are increased and the LUMO energies are decreased in turn since the size of the thiophene-containing increases. Therefore, the E_g_ of the dyes are reduced in turn. Compared the C1-*n* and C2-*n* (*n* = 1–4) dyes with giving *n*, the corresponding π-conjugated linker of C1-*n* dyes have one vinylene group more than that of C2-*n* dyes, though they have same electron donor and acceptor groups, and then the HOMO energies of C1-*n* are more positive, the LUMO energies of C1-*n* are more negative, and the *E*_g_ of C1-*n* is smaller. The comparison of the dyes with bithiophene and terthiophene in conjugate bridges suggests that introducing rigid planar dithieno-[3,2-b;2′,3′-d] thiophene in conjugate bridges generate lower HOMO energies and broader E_g_. From the data of C1-2 and C1-5, it can be found that inserting vinylene group in the middle of bithiophene elevate HOMO about 0.09 eV in gas phase (0.05 eV in solvent), decline LUMO about 0.09 eV in gas phase (0.06 eV in solvent), and thus reduce *E*_g_ about 0.17 eV in gas phase (0.11 eV in solvent). The comparison of C2-1 and C3-1 indicates that substitution of hydrogen with phenyl increase HOMO about 0.08 eV in solvent, and the reduced *E*_g_ by about 0.25 eV.

### 3.3. Absorption and Emission Spectra: UV-Vis and Fluorescence Spectra

The electronic absorption spectra of tetrahydroquinoline dyes C1-1, C1-2, C1-3, C1-5, C2-1, C2-2, and C3-1 were measured in ethanol solution, and the absorption spectra of the dyes C1-4, C2-3, and C2-4 were measured in DMF solution. The experimental and calculated electronic absorption λ_max_ (nm/eV), as well as the λ_max_ errors (nm/eV) between the experiment and the calculation are listed in Table 4. It can be found that the calculated results agree well with that of experiment. So, the results of CAM-B3LYP functional combined with PCM method are reliable for analyzing excited state properties of tetrahydroquinoline dyes. Apparently, with the elongating conjugate bridge by thiophene units, the corresponding λ_max_ has a red-shift, which is favorable for matching the solar radiation spectra. The corresponding oscillator strength also increases in the order because the longer conjugate bridge extends the overlap of HOMO and LUMO.

To obtain the microscopic information about the electronic transitions, we check the corresponding MO properties. The absorptions in visible and near-UV region are the most important regions for photo to current conversion, so only the singlet→singlet transitions of the absorption bands with the wavelength longer than 300 nm and the oscillator strength larger than 0.1 are listed in Table 5. The isodensity plots of the frontier MOs that related to the absorptions in visible and near-UV region are presented in Table 6. The MOs indicate that the HOMO-1 of the dyes are delocalized in the molecules, and the HOMOs are mainly contributed from the framework of quinoline moiety to conjugate bridge groups, whereas the LUMOs and LUMO + 1 mainly locate on the acceptor cyanoacetic acid moieties and conjugate bridge groups. In addition, the molecular orbital overlaps of HOMO and LUMO are highly coupled to the π-conjugated linker. For these dyes, the maximum absorptions in UV/vis spectra are dominated by HOMO→LUMO π→π* transitions. Furthermore, the overlap between HOMO and LUMO of the dyes suggest the maximum absorptions have some local excited transitions in conjugate bridge. While the relocations of the HOMOs and LUMOs in the dyes support that the transitions at maximum absorptions have intramolecular charge transfer (IMCT) character. The further MO analysis indicates that the quinoline groups are effective chromophores in IMCT, and then they play an important role in the sensitization of DSC.

**Figure 6:**
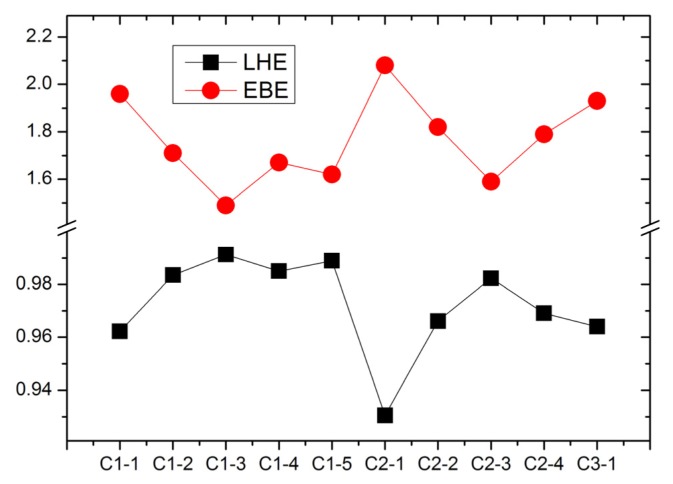
The calculated light harvesting efficiencies (LHE) and the exciton binding energies (EBE, in eV) of tetrahydroquinoline dyes.

For the dye sensitizers with good performance in DSCs, the LHE are expected to be as high as possible to increase the photocurrent if the excited processes have CT character. LHE can be calculated as [73]:

(1) $$\text{LHE}=1-10^{-\text{A}}=1-10^{-f}$$

where *A* is the absorption coefficient and *f* is oscillator strength of the excited state associated to the $\lambda_{\text{max}}^{\left(1\right)}$. The calculated LHE of tetrahydroquinoline dyes are presented in Figure 6. It can be found that C1-3 and C2-3 have the largest LHE in C1-*n* (*n* = 1–5) and C2-*n* (*n* = 1–4) series, respectively. In addition, the elongating of conjugate bridge generates the larger oscillator strength and LHE because of the enhanced overlap between the ground states (mainly contributed by HOMO) and the first excited states (mainly contributed by LUMO). So, extending the conjugate bridge in D-π-A organic dye is an effective way to increase the harvest of solar light.

The EBE, as an important quantity for the efficiency of excitonic solar cells, determines the charge separation in solar cells [42]. The EBE can be calculated as the difference between the electronic and optical band gap energies [74]. The electronic band gap is calculated as the energy difference between the HOMO and LUMO levels, while the first excitation energy is adopted as the optical gap [42,75]. The calculated EBE (in eV) of tetrahydroquinoline dyes are shown in Figure 6. It indicates that the elongating of conjugate bridge generates the decreasing of EBE, which is favorable for photo-to-current energy conversion via the dissociation of a bound exciton (hole/electron pair). Furthermore, the smallest EBE of C1-3 and C2-3 dyes suggest the exciton can dissociate more effectively than that in other dyes. More interestingly, the figure reveals that the EBE and the LHE are inversely correlated, implying that the dyes with lower EBE produce more efficient light harvesting. This agrees with the relationship between the EBE and quantum yield [42].

Concerning fluorescence, the calculated and experimental emission maxima λ and Stokes shift (SS) for the tetrahydroquinoline dyes are listed in Table 7. For most of the dyes, except C1-1 and C2-1, the computed emission maxima are in good agreement with the experimental data because the discrepancy is lower than 0.3 eV. Particularly, for C2-3 and C1-4, a quantitatively agreement with the experimental results is obtained since the errors are about 0.03 and 0.08 eV, respectively. While in the case of C1-1 and C2-1, the errors (0.34 and 0.45 eV, respectively) are larger than that of others. This is probably to be ascribed to the neglect of direct solute-solvent interactions [76]. After state specific correction, the calculated emission maxima of C1-1 and C2-1 dyes are about 2.14 and 2.41 eV, respectively, and therefore the errors of emission maxima are reduced to 0.22 and 0.32 eV, respectively. Compared with emission maxima, the SS has similar errors. It can be found that, except C1-3, elongating conjugate bridge generates red shift of emission maxima, larger oscillator strength, and increasing of SS due to the enhanced flexibility by the longer conjugate linker, allowing for a larger structural relaxation from the excited state S_1_ to the ground state S_0_.

Radiative lifetimes for spontaneous emission from S_1_ were computed from fluorescene energies (*E_fluo_*) and oscillator strength, which represents the transition probability (*f* in a.u.) as [76]:

(2) $$\tau =\frac{2\pi {ɛ}_{0}{\text{m}}_{\text{e}}\hbar^{2}{\text{c}}^{3}}{{\text{e}}^{4}{\left({\text{E}}_{\text{fluo}}\right)}^{2}\text{f}}$$

where, *m*_e_ is the electron mass, ɛ_0_ is the vacuum permittivity, *h̄* is the reduced Plank constant, *c* is the speed of light, and *e* is the elementary charge. To investigate the role of conjugate bridge in radiative lifetime, rather than accurate reproduce the radiative lifetime, we define relative radiative lifetime (*τ*_rel_) of the dye molecule by assuming C1-1 radiative lifetime (ι_C1–1_) as reference value:

(3) $$\tau_{\text{rel}}=\frac{\tau }{\tau_{\text{C}1-1}}=\frac{{\left(E_{\text{fluoC}1-1}\right)}^{2}f_{\text{C}1-1}}{{\left(E_{\text{fluoi}}\right)}^{2}f_{\text{i}}}$$

where *E*_fluoC1–1_, *E*_fluoi_, *f*_C1–1_, and *f*_i_ are fluorescene energies and oscillator strength of C1-1 and the selected dye molecule, respectively. The calculated relative radiative lifetime of the tetrahydroquinoline dyes are listed in Table 6. Similar to oscillator strength, the relative radiative lifetime is increasing with the elongating conjugate bridge by thiophene unit. The shortest lifetime of C1-4 and C2-4 may be ascribed to their planar conjugation (by dithienothiophene group), favoring charge recombination.

### 3.4. The Driving Force of Electron Injection and Dye Regeneration

The electron injection from the excited dyes to semiconductor conduction band and the dye regeneration processes can be described as CT reaction. The driving force, which is defined as the potential difference between the oxidized dyes and the electrolyte, determines the yield of electron collection and dye regeneration. Furthermore, the oxidation potential of the dyes must be more positive than that of electrolyte, thus ensuring enough driving force for a fast and efficient regeneration of the dye cation radical and avoiding the geminate charge recombination between oxidized dye sensitizers and the nanocrystalline TiO_2_ film where the photo-excited electrons are injected. In terms of the Marcus theory for electron transfer [77], the CT rate constants can be affected by free energy change related to the reaction. The free energy change for electron injection ( Δ*G^inject^* ) affects the electron injection rate and therefore the *J_sc_* in DSCs. Δ*G^inject^* can be viewed as the electron injection driving force [44]. According to Preat’s method [73], assuming the electron injection occurs from the unrelaxed excited state of the dye, the Δ*G^inject^* can be calculated by the following Equation:

(4) $$\Delta G^{inject}=E_{OX}^{dye*}-E_{CB}^{SC}$$

where $E_{OX}^{dye*}$ is the oxidation potential of the dye in the excited state and $E_{CB}^{SC}$ is the reduction potential of the conduction band of the semiconductor. The reported $E_{CB}^{SC}=4.0\;\text{eV}$ for TiO_2_[78] was adopted in this work. The $E_{OX}^{dye*}$ can be calculated as following [79]:

(5) $$E_{OX}^{dye*}=E_{OX}^{dye}-\lambda_{\text{max}}$$

in which $E_{OX}^{dye}$ is the redox potential of the ground state and *λ*_max_ is the absorption maximum with IMCT character. Whereas the free energy change of dye regeneration ( $\Delta G_{\mathrm{dye}}^{\mathrm{regen}}$ ) can affect on the rate constant of redox process between the oxidized dyes and electrolyte. The $\Delta G_{\mathrm{dye}}^{\mathrm{regen}}$ can be calculated as

(6) $$\Delta G_{dye}^{regen}=E_{OX}^{dye}-E_{redox}^{electrolyte}$$

where $E_{\mathrm{redox}}^{\mathrm{electrolyte}}$ is the redox potential of electrolyte. The $E_{\mathrm{redox}}^{\mathrm{electrolyte}}$ of commonly used redox couple iodide/triiodide is about 4.85 eV (0.35 V *vs.* NHE) [80]. The calculated $E_{OX}^{dye},E_{OX}^{dye*}$, Δ*G^inject^*, and $\Delta G_{\mathrm{dye}}^{\mathrm{regen}}$ for tetrahydroquinoline dyes are listed in Table 8.

In terms of the calculated results, the negative Δ*G^inject^* of the tetrahydroquinoline dyes indicates that the excited state with IMCT character lies above the TiO_2_ conduction band edge. For C1-*n* (*n* = 1–5) dyes, the absolute value of Δ*G^inject^* for C1-3 is larger than that of other dyes, and the absolute value of Δ*G^inject^* increases with the elongating of conjugate bridges with thiophene units. The Δ*G^inject^* of C1-4 which has dithienothiophene in conjugate linker is almost as same as that of C1-3. In addition, the data of C1-5 means that inserting a vinyl unit between bithiophene in conjugate bridges induces a slight decreasing of Δ*G^inject^* (about 0.02 eV). Furthermore, with elongating conjugate bridges, the $\Delta G_{\mathrm{dye}}^{\mathrm{regen}}$ of the dyes decrease in the corresponding order. So, introducing longer conjugate bridges in dyes can suppress electron recombination. For C2-*n* (*n* = 1–4) dyes, the variation of Δ*G^inject^* is only about 0.05 eV (from -0.59 to -0.64 eV). The C2-3 and C2-4 have largest absolute value of Δ*G^inject^*, and the dependence of $\Delta G_{\mathrm{dye}}^{\mathrm{regen}}$ on the number of thiophene unit in conjugate bridge is similar to that of C1-*n* (*n* = 1–4) dyes. Comparing the data of C1-*n* and C2-*n* (*n* = 1–4), it is found that inserting vinyl units between quinoline and thiophene generate a reduction of absolute value Δ*G^inject^* of and $\Delta G_{\mathrm{dye}}^{\mathrm{regen}}$.

## 4. Conclusions

Conjugate bridge of organic dye sensitizer for solar cells can modify their electronic structure based properties, and therefore the performance in DSCs. To understand the role of conjugate bridge in the modification of electronic structure based properties, the computations of the geometries and electronic structures for 10 kinds of tetrahydroquinoline dyes were performed using DFT, and the electronic absorption and fluorescence properties were investigated via TDFT with PCM for solvent effects. The population analysis, molecular orbital energies, radiative lifetimes, EBE, LHE, Δ*G^inject^*, and $\Delta G_{\mathrm{dye}}^{\mathrm{regen}}$ were also addressed.

The population analysis of NBO results indicates that more charges populated in acceptor groups correspond to larger *V*_oc_. This means introducing strong electron-withdrawing group in the acceptor of dye sensitizer is a possible way to increase *V*_oc_ of DSC. The absorption properties and MO analysis indicate that the quinoline groups are effective chromophores in IMCT, and they take the function of electron injection in DSCs.

The elongating of a conjugate bridge generates the larger oscillator strength, higher LHE, larger the absolute value of Δ*G^inject^*, and longer the relative radiative lifetime, as well as the decreasing of EBE and the $\Delta G_{\mathrm{dye}}^{\mathrm{regen}}$. So extending the conjugate bridge with thiopene units in D-π-A organic dye is an effective way to increase the harvest of solar light, also it is favorable for fast electron injection due to larger Δ*G^inject^*. While the inversely correlated relationship between EBE and LHE imply that the dyes with lower EBE have more efficient light harvesting. The decreasing of $\Delta G_{\mathrm{dye}}^{\mathrm{regen}}$ with the elongating conjugate bridge implies that introducing longer conjugate bridges can suppress electron recombination.

## Figures and Tables

**Table 1:** The chemical structures of the tetrahydroquinoline dyes.

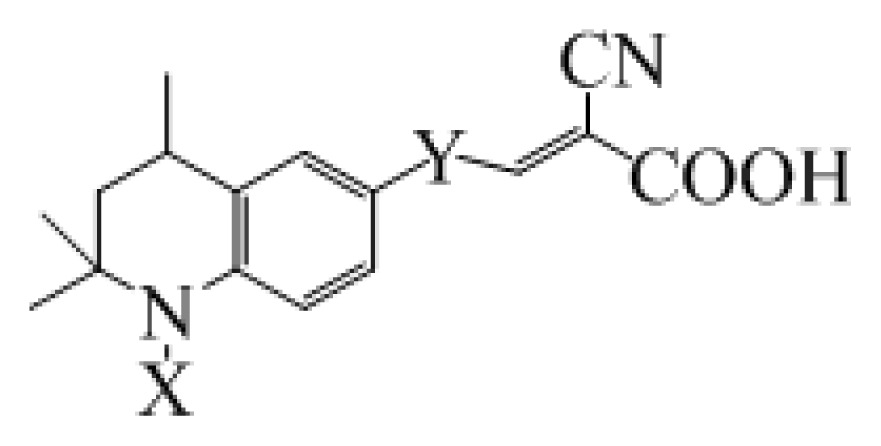					
Dyes	X=	Y=	Dyes	X=	Y=
C1-1	CH_3_	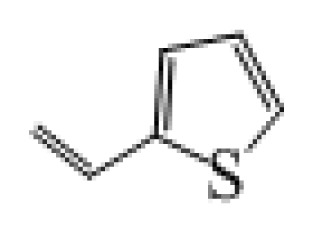	C2-1	CH_3_	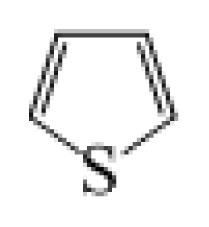
C1-2	CH_3_	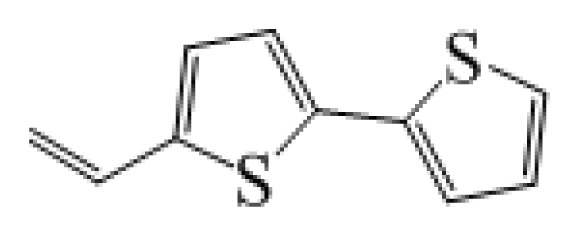	C2-2	CH_3_	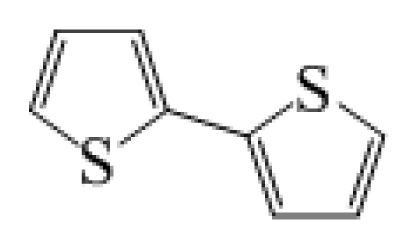
C1-3	CH3	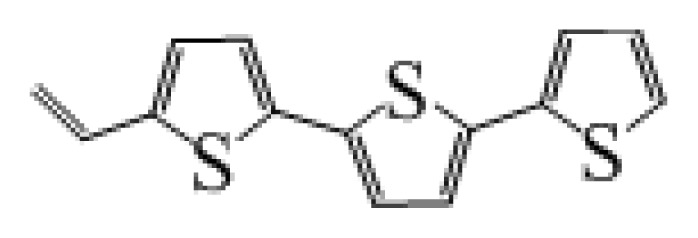	C2-3	CH_3_	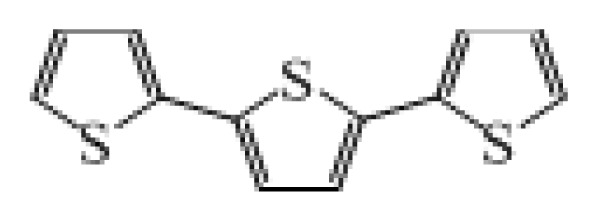
C1-4	CH_3_	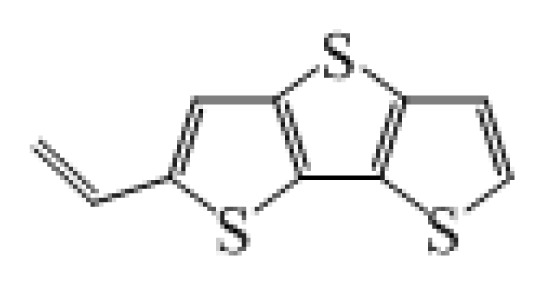	C2-4	CH_3_	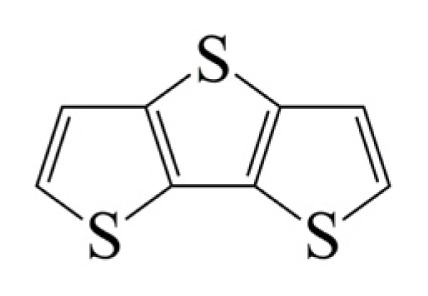
C1-5	CH_3_	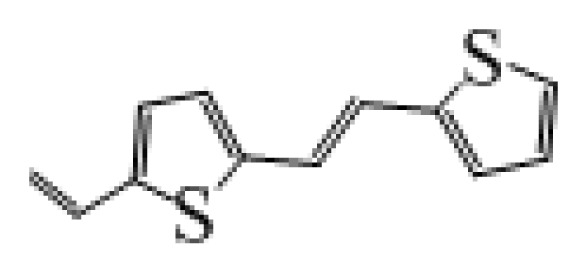	C3-1	Ph	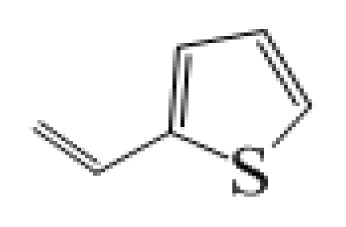

**Table 2:** Experimental absorption maxima, the computed excitation energies λ_max_ (nm/eV) and oscillator strength *f* of the lowest excited state for the dye C1-1 in ethanol solution, provided by PCM-TDDFT/ 6-31G (d,p).

	PBE0	CAM-B3LYP	LC-ωPBE	ωB97X	M062X	Experiment
λ_max_	569/2.18	472/2.63	395/3.14	414/2.99	474/2.62	468/2.65
*f*	1.3604	1.4230	1.4189	1.4148	1.3526	–

**Table 3:** The charge populations of the ground state (S_0_) for tetrahydroquinoline dyes.

Dyes	Donating group	π-Conjugated linker	Acceptor group
C1-1	0.155	0.025	−0.180
C1-2	0.131	0.039	−0.170
C1-3	0.115	0.047	−0.162
C1-4	0.138	0.027	−0.164
C1-5	0.126	0.043	−0.169
C2-1	0.202	−0.015	−0.187
C2-2	0.163	0.009	−0.172
C2-3	0.149	0.015	−0.164
C2-4	0.171	−0.005	−0.165
C3-1	0.151	0.029	−0.180

**Table 4:** The experimental and calculated electronic absorption λ_max_ (nm/eV), as well as the λ_max_ errors (nm/eV) between the experiment and the calculation of tetrahydroquinoline dyes.

Dyes	Experimental λ_max_	Calculated λ_max_	error
C1-1	468/2.65	472/2.63	6/0.02
C1-2	472/2.63	483/2.55	11/0.08
C1-3	475/2.61	476/2.61	1/0.00
C1-4	467/2.65	470/2.64	3/0.01
C1-5	492/2.52	499/2.48	7/0.04
C2-1	441/2.81	434/2.86	7/0.05
C2-2	462/2.68	457/2.71	5/0.03
C2-3	455/2.72	460/2.69	5/0.03
C2-4	444/2.79	441/2.81	3/0.02
C2-5	470/2.64	465/2.67	5/0.03

**Table 5:** The calculated excitation energies (eV/nm), electronic transition configurations and oscillator strengths (*f*) for tetrahydroquinoline dyes in solution (CAM-B3LYP/6-31G(d,p)).

Dyes	State	Configurations composition with |coeff.| > 0.2 (transition oribitals)	Excitation energy	*f*
C1-1	1	0.6617(H → L)	2.63/472	1.4230
	2	0.6370(H − 1 → L)	3.97/313	0.1305

C1-2	1	0.6080(H → L); -0.2892(H − 1 → L)	2.55/483	1.7838
	2	0.5426(H − 1 → L); −0.3226(H → L + 2); −0.2109(H − 3 → L)	3.60/344	0.0022
	3	0.5499(H → L + 1); 0.2860(H → L); 0.2298(H − 1 → L)	3.90/318	0.3394

C1-3	1	0.5272(H → L); −0.3657(H − 1 → L); −0.2453(H → L + 1)	2.61/476	2.0586
	2	0.4624(H → L + 1); −0.4117(H − 1 → L); 0.2571(H − 2 → L)	3.37/367	0.2063
	3	0.4336(H → L); 0.3773(H → L + 1); 0.2856(H − 1 → L)	3.70/335	0.3175

C1-4	1	0.6138(H → L); −0.2753(H − 1 → L)	2.64/470	1.8212
	2	0.5204(H − 1 → L); −0.3108(H → L + 1); 0.2258(H − 2 → L)	3.65/340	0.1647
	3	0.6496(H − 2 → L)	3.83/324	0.1734
	4	0.5542(H → L + 1);0.2836(H → L); 0.2423(H − 1 → L)	3.98/312	0.3454

C1-5	1	0.5885(H → L); −0.3119(H − 1 → L)	2.48/499	1.9599
	2	0.5146(H − 1 → L); 0.3505(H → L + 1); 0.2427(H − 2 → L)	3.43/361	0.0147
	3	0.5168(H → L + 1); −0.3192(H → L); −0.2396(H − 1 → L)	3.74/331	0.3021

C2-1	1	0.6732(H → L); −0.1707(H − 1 → L)	2.86/434	1.1582

C2-2	1	0.6210(H → L); −0.2866(H − 1 → L)	2.71/457	1.4695
	2	0.5942(H − 1 → L); 0.2351(H → L)	3.80/326	0.0264

C2-3	1	0.5470(H → L); −0.3771(H − 1 → L)	2.69/460	1.7513
	2	0.4771(H − 1 → L); 0.3489(H → L + 1); 0.26105(H − 2 → L); 0.2354(H → L)	3.57/347	0.0684
	3	0.4921(H → L + 1); −0.3547(H → L + 1); −0.20695(H − 1 → L)	3.89/319	0.3308

C2-4	1	0.6227(H → L); −0.2849(H − 1 → L)	2.81/441	1.5097
	2	0.5318(H − 2 → L); −0.3719(H − 1 → L)	3.80/327	0.0072
	3	0.4731(H − 1 → L);0.4340(H − 2 → L)	3.93/316	0.2227

C3-1	1	0.6618(H → L)	2.67/465	1.4440
	2	0.6327(H − 1 → L)	4.00/310	0.1103

**Table 6:** Isodensity plots (isodensity contour = 0.02 a.u.) of the frontier molecular orbitals of the tetrahydroquinoline dyes.

Dyes	HOMO − 1	HOMO	LUMO	LUMO + 1
**C1-1**	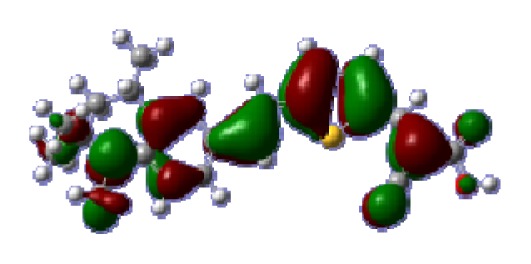	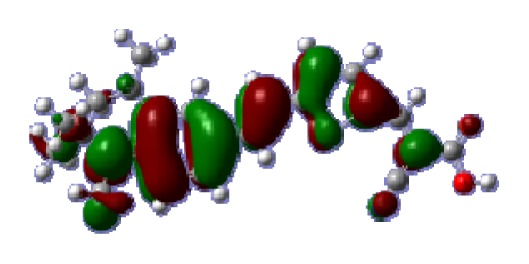	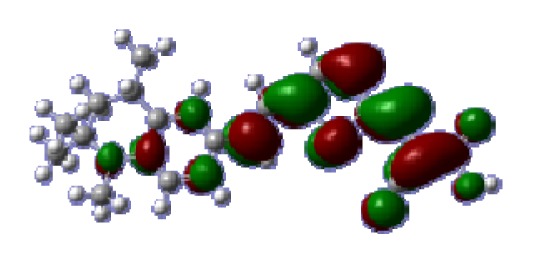	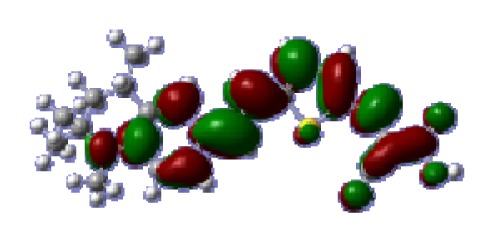
**C1-2**	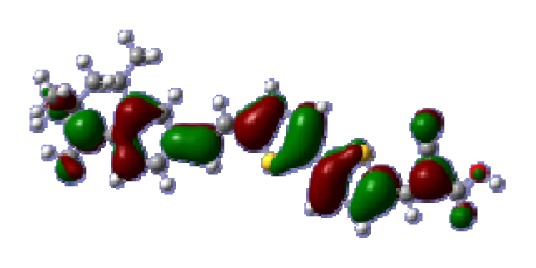	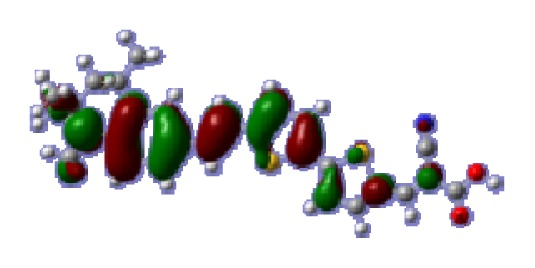	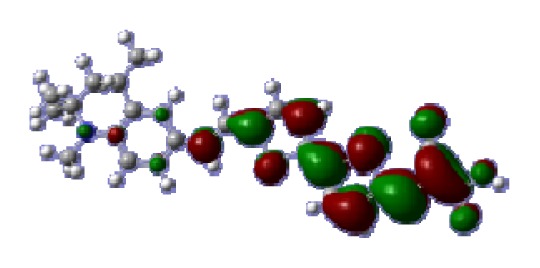	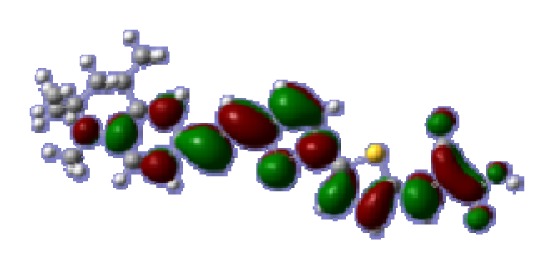
**C1-3**	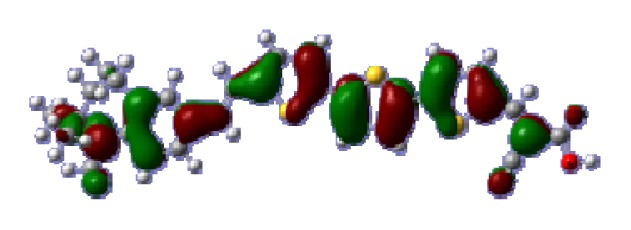	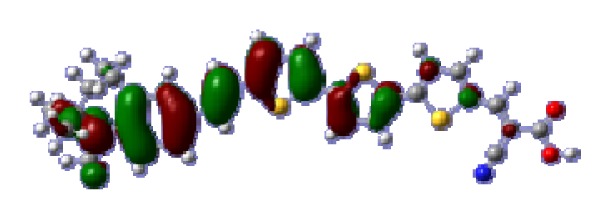	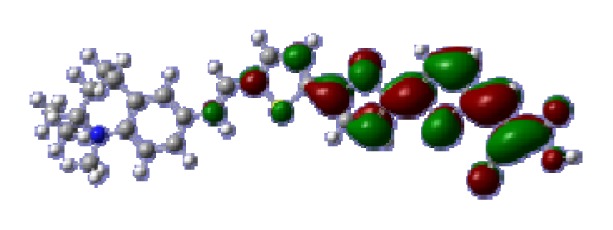	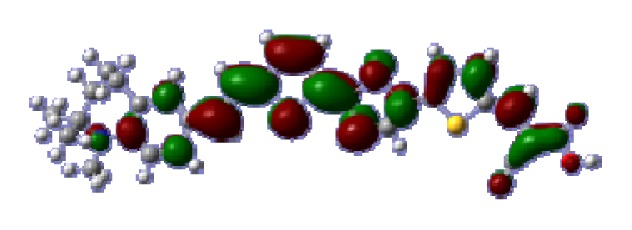
**C1-4**	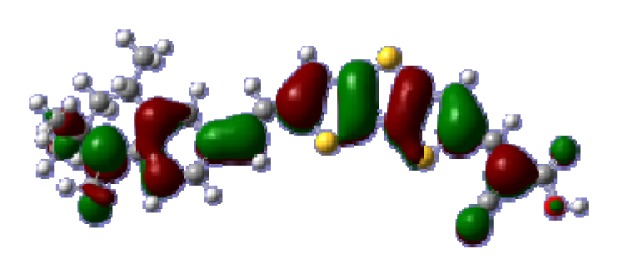	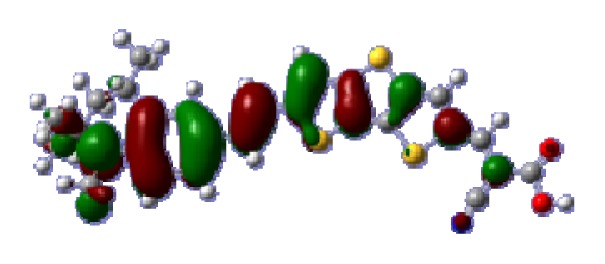	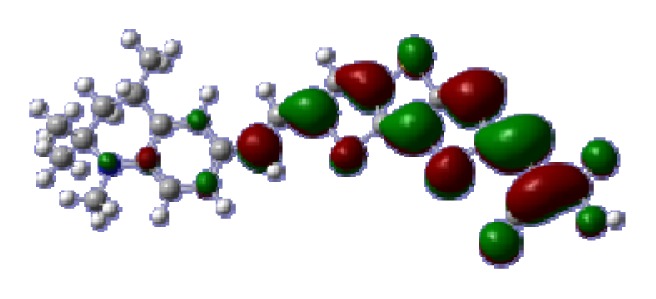	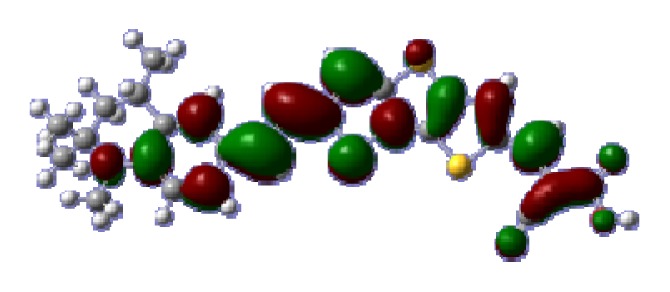
**C1-5**	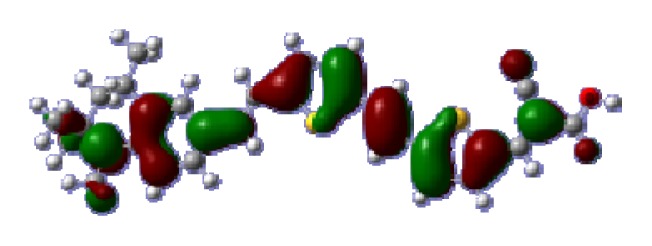	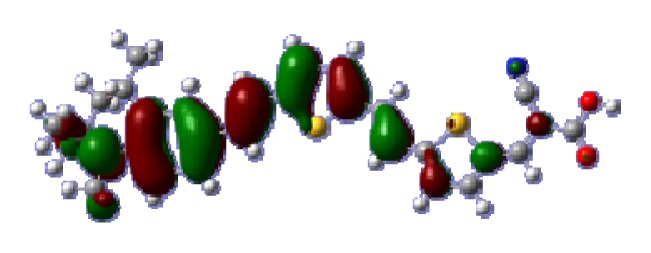	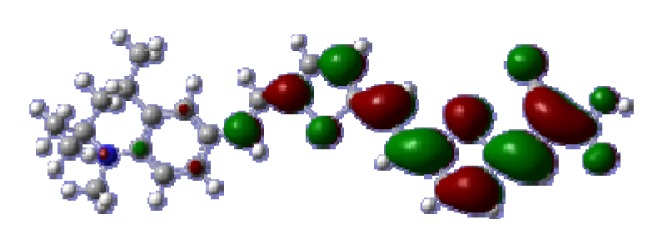	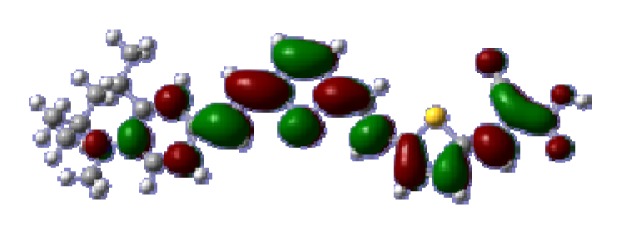
**C2-1**	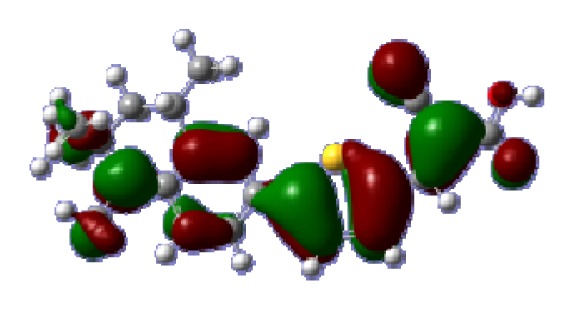	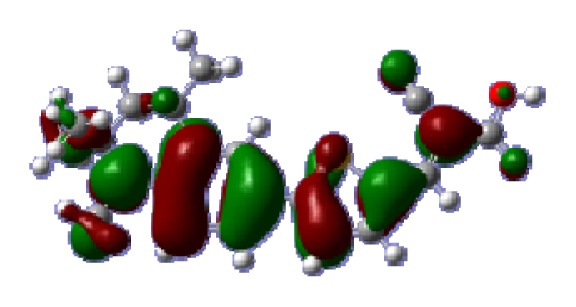	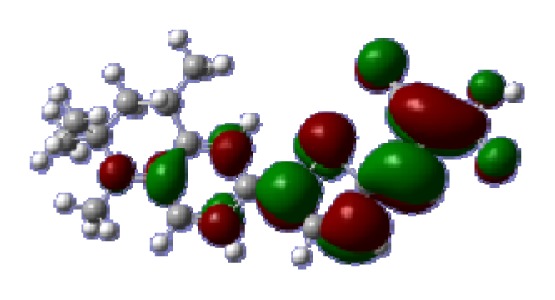	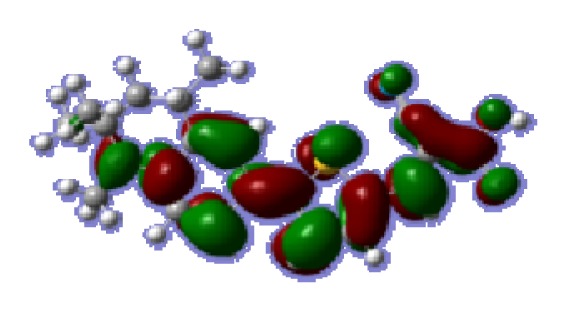
**C2-2**	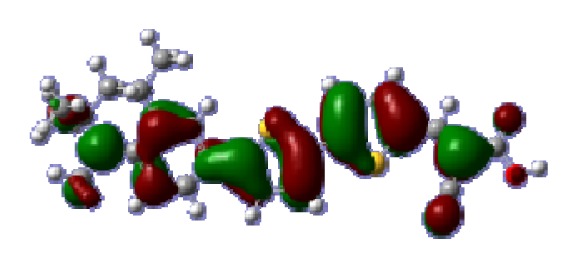	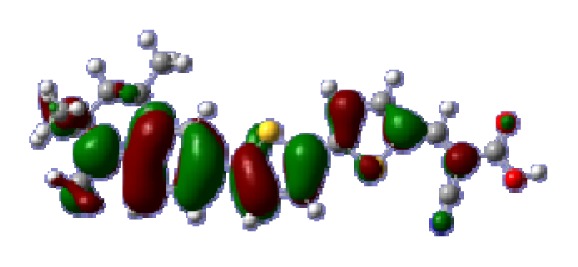	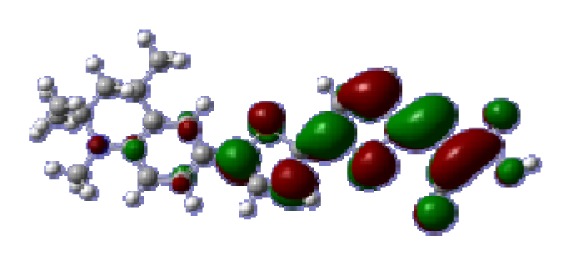	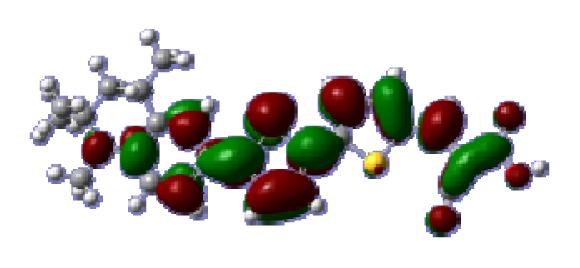
**C2-3**	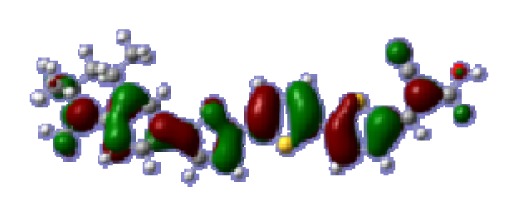	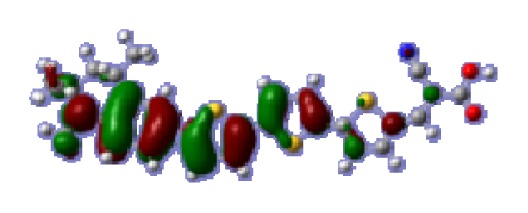	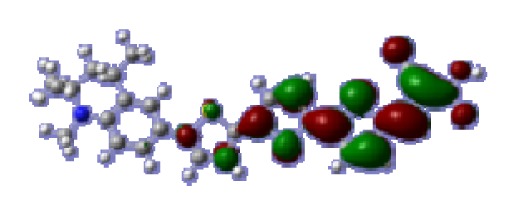	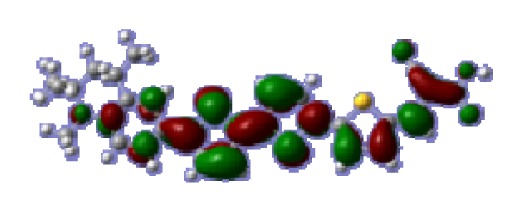
**C2-4**	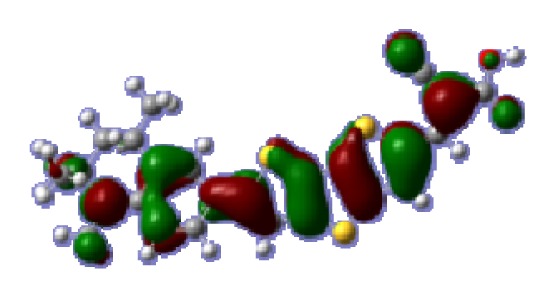	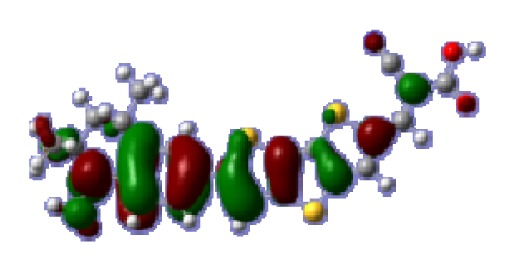	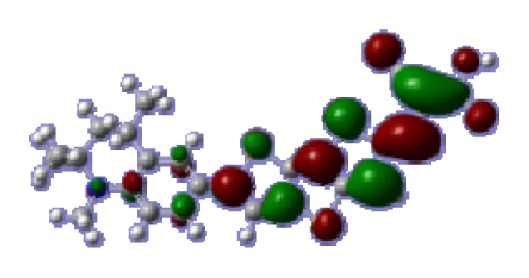	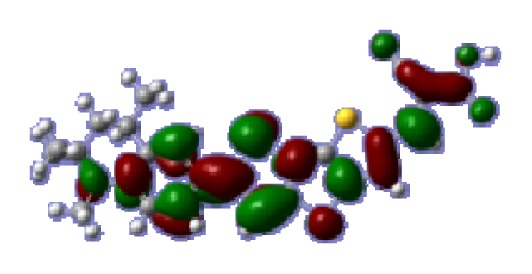
**C3-1**	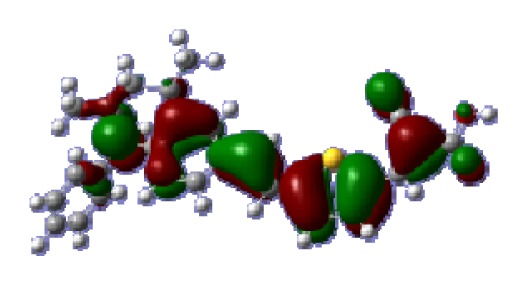	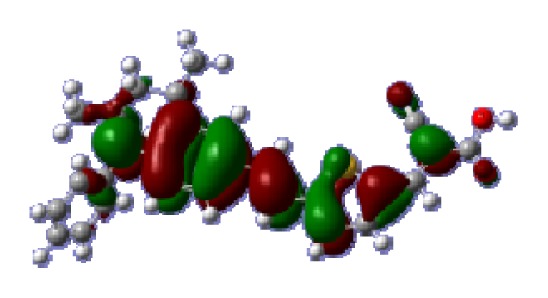	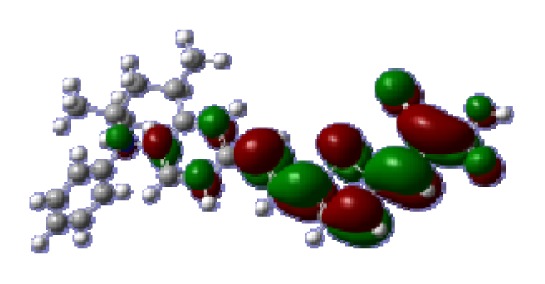	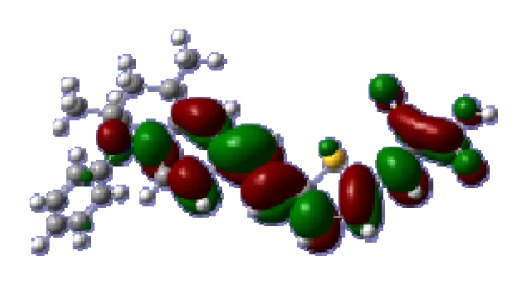

**Table 7:** Fluorescence maxima (λ in nm/eV), oscillator strength (*f* in a.u.) and relative radiative lifetimes ( *τ*_rel_ ) of tetrahydroquinoline dyes in solution with respect to the experimental results. The corresponding Stokes shifts (SS, in eV) are also reported.

Dyes	Computed			Experimental a		

	λ	*F*	*ι*_rel_	SS	λ	SS
C1-1	549/2.26	1.6166	1.00	0.37	647/1.92	0.73
C1-2	640/1.94	2.1251	1.03	0.61	670/1.85	0.78
C1-3	701/1.77	2.3572	1.12	0.84	615/2.02	0.59
C1-4	618/2.01	2.1758	0.94	0.63	643/1.93	0.72
C1-5	687/1.80	2.3787	1.07	0.68	656/1.89	0.63
C2-1	488/2.54	1.2562	1.02	0.32	594/2.09	0.72
C2-2	583/2.13	1.7457	1.04	0.58	652/1.90	0.78
C2-3	662/1.87	2.1131	1.18	0.82	652/1.90	0.82
C2-4	558/2.22	1.7799	0.94	0.59	612/2.03	0.76
C3-1	551/2.25	1.6510	0.99	0.42	635/1.95	0.69

a the experimental results were reported in [47].

**Table 8:** The calculated redox potential (in eV), Δ*G^inject^* (in eV), and $\Delta G_{\mathrm{dye}}^{\mathrm{regen}}$ (in eV) for the tetrahydroquinoline dyes in the solution.

Dye	$E_{OX}^{dye}$	$E_{OX}^{dye*}$	Δ*G^inject^*	$\Delta G_{\mathrm{dye}}^{\mathrm{regen}}$
C1-1	6.09	3.46	−0.54	1.24
C1-2	5.97	3.42	−0.58	1.12
C1-3	5.96	3.35	−0.65	1.11
C1-4	6.00	3.36	−0.64	1.15
C1-5	5.92	3.44	−0.56	1.07
C2-1	6.25	3.39	−0.61	1.40
C2-2	6.12	3.41	−0.59	1.27
C2-3	6.05	3.36	−0.64	1.20
C2-4	6.17	3.36	−0.64	1.32
C3-1	6.17	3.50	−0.50	1.32
